# Eleven Candidate Susceptibility Genes for Common Familial Colorectal Cancer

**DOI:** 10.1371/journal.pgen.1003876

**Published:** 2013-10-17

**Authors:** Alexandra E. Gylfe, Riku Katainen, Johanna Kondelin, Tomas Tanskanen, Tatiana Cajuso, Ulrika Hänninen, Jussi Taipale, Minna Taipale, Laura Renkonen-Sinisalo, Heikki Järvinen, Jukka-Pekka Mecklin, Outi Kilpivaara, Esa Pitkänen, Pia Vahteristo, Sari Tuupanen, Auli Karhu, Lauri A. Aaltonen

**Affiliations:** 1 Department of Medical Genetics, Genome-Scale Biology Research Program, University of Helsinki, Helsinki, Finland; 2 Institute of Biomedicine, Genome-Scale Biology Research Program, University of Helsinki, Helsinki, Finland; 3 Science for Life Center, Department of Biosciences and Nutrition, Karolinska Institutet, Stockholm, Sweden; 4 Department of Surgery, Helsinki University Hospital, Helsinki, Finland; 5 Department of Surgery, Jyväskylä Central Hospital, University of Eastern Finland, Jyväskylä, Finland; University of Washington, United States of America

## Abstract

Hereditary factors are presumed to play a role in one third of colorectal cancer (CRC) cases. However, in the majority of familial CRC cases the genetic basis of predisposition remains unexplained. This is particularly true for families with few affected individuals. To identify susceptibility genes for this common phenotype, we examined familial cases derived from a consecutive series of 1514 Finnish CRC patients. Ninety-six familial CRC patients with no previous diagnosis of a hereditary CRC syndrome were included in the analysis. Eighty-six patients had one affected first-degree relative, and ten patients had two or more. Exome sequencing was utilized to search for genes harboring putative loss-of-function variants, because such alterations are likely candidates for disease-causing mutations. Eleven genes with rare truncating variants in two or three familial CRC cases were identified: *UACA*, *SFXN4*, *TWSG1*, *PSPH*, *NUDT7*, *ZNF490*, *PRSS37*, *CCDC18*, *PRADC1*, *MRPL3*, and *AKR1C4*. Loss of heterozygosity was examined in all respective cancer samples, and was detected in seven occasions involving four of the candidate genes. In all seven occasions the wild-type allele was lost (*P* = 0.0078) providing additional evidence that these eleven genes are likely to include true culprits. The study provides a set of candidate predisposition genes which may explain a subset of common familial CRC. Additional genetic validation in other populations is required to provide firm evidence for causality, as well as to characterize the natural history of the respective phenotypes.

## Author Summary

Many individuals with a family history of colorectal cancer have no detectable germline mutation in the known cancer predisposing genes. We aimed to identify novel susceptibility genes for this common phenotype by performing exome sequencing on 96 independent cases with familial colorectal cancer. Eighty-six patients had one affected first-degree relative, and ten patients had two or more. None of the patients had a previous diagnosis of a hereditary syndrome. We focused our search on genes with rare variants, predicted to truncate the protein product, since these are likely candidates for disease predisposition. Using this approach we identified truncating germline variants in eleven genes, present in two or three independent familial colorectal cancer cases. We analyzed the respective tumor DNAs and found loss of the wild-type allele in seven out of seven occasions, involving four genes. No tumor showed loss of the mutant allele which provides us with additional evidence for disease causality. Further studies are required to provide firm evidence for pathogenicity. Genetic knowledge on confirmed predisposing genes can ultimately be translated into tools for cancer prevention and early diagnosis in individuals carrying predisposition alleles.

## Introduction

Colorectal cancer (CRC) (MIM 114500) is a major cancer type, with over one million new cases diagnosed worldwide each year. It is the third most common malignancy [1], and the second most common cause of cancer mortality [2]. Inherited factors are estimated to play a crucial role in at least one third of all CRC cases [3]. However, high-penetrance mutations in known CRC predisposing genes, such as the mismatch repair (MMR) genes, *APC*, *MUTYH* (*MYH*), *SMAD4*, *BMPR1A*, *STK11/LKB1*, *PTEN*, *AXIN2*, *POLE*, and *POLD1* explain only around 5% of these cases [4]–[6].

There are a few examples of rare variants in CRC predisposing genes conferring moderate or low carrier risk, such as *APC* (I1307K) [7], *BLM* [8] and *GALNT12* [9]. Of these, the *APC* I1307K variant has been most extensively studied and occurs almost exclusively in the Ashkenazi Jewish population [7]. In addition to these, genome-wide association (GWA) studies have identified common low-penetrance variants at approximately 20 genomic loci associated with CRC susceptibility. However, the identified common variants at these loci exert only a modest effect on CRC risk [10]–[12].

Unknown variants of moderate or low penetrance are likely to explain at least part of the missing heritability in CRC. CRC families with few affected individuals are an attractive patient group to search for such genetic factors, but tools for such work have been poor. These families are relatively common but too small for linkage analyses, and the culprit variants are likely to be too diverse and rare to be detected in GWA studies. One approach has been to study the additive contribution of low-penetrance variants on familial risk. A previous study has estimated that ten known low-penetrance CRC variants collectively explain around 9% of the variance in familial risk [13]. Advances in sequencing technologies have made exome sequencing a feasible approach to search for rare coding variants of varying penetrance. In this study, we aimed at identifying variants predisposing to common familial CRC by performing exome sequencing on 96 independent familial CRC cases derived from a consecutive collection of unselected patients. Here, familial CRC is characterized as having at least one first-degree relative diagnosed with CRC; indeed the great majority of the 96 familial cases displayed only one first-degree relative with CRC. All patients were from Finland, known for its relatively homogenous population [14], [15]. This empowers the analysis since affected individuals are more likely to share ancestral predisposition mutations and haplotypes, stemming from a handful of founders. To our knowledge, this is the largest effort to date where exome sequencing has been applied to familial forms of cancer to identify novel predisposing genes.

## Results

### Exome sequencing

**Figure 1:**
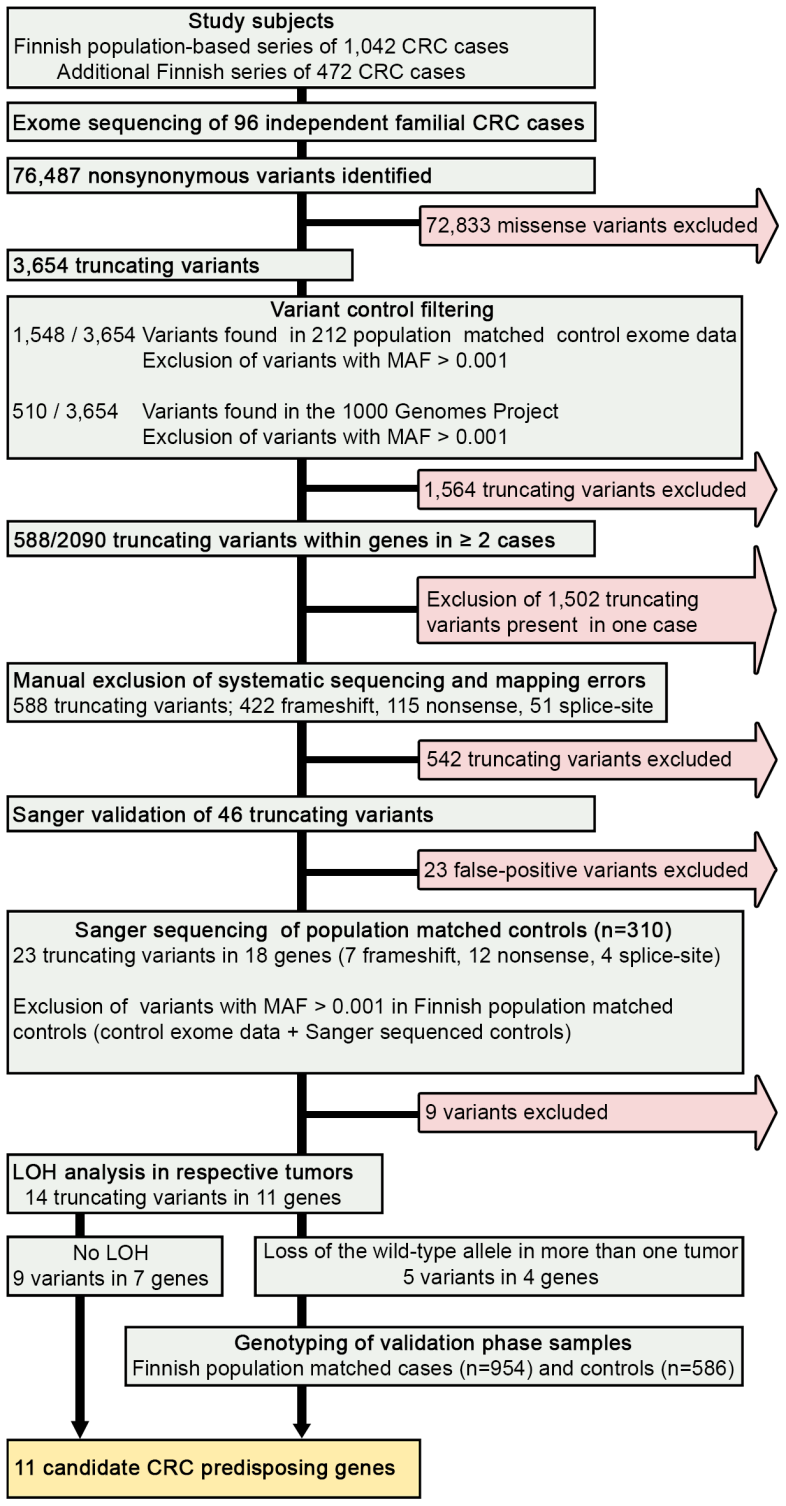
Schematic representation of the overall study design. We performed exome sequencing analysis of germline DNA from 96 independent familial CRC cases. Initially, quality, frequency and control filtering were applied to the exome data. Next, genes with putative truncating loss-of-function variants in at least 2/96 cases were validated by Sanger sequencing. Confirmed truncating variants were then screened in Finnish population matched controls. Loss of heterozygosity was analyzed in the respective tumor tissues. Variants in genes showing loss of the wild-type allele in tumor tissue were genotyped in a set of validation phase samples. Overall, 11 novel candidate CRC predisposing genes were identified. CRC, colorectal cancer; MAF, minor allele frequency; LOH, loss of heterozygosity.

Exome sequencing analysis was performed on germline DNA from 96 independent familial CRC cases. The clinical and histopathological features of the cases are summarized in Table 1 and in more detail in Table S1. The average read depth attained for target regions was 43 and at least 86% of the captured target regions were covered by four or more sequence reads for all the samples. We identified a total of 76,487 nonsynonymous variants in the exome data (Figure 1). Sequence data were first evaluated for known predisposing genes (*MLH1*, *MSH2*, *MSH6*, *PMS2*, *APC*, *MUTYH*, *SMAD4*, *BMPR1A*, *LKB1/STK11*, *PTEN*, *AXIN2*, *POLE*, and *POLD1*). No clear pathogenic mutations were found in these genes. The following missense variants were identified (not confirmed by Sanger sequencing); *MSH6* c.2800G>C p.D934H, and *PTEN* c.1016C>A P339Q. However, the patients did not present typical clinical phenotypes; in the case of the *MSH6* variant the tumor did not display microsatellite instability and in the case of the *PTEN* variant patient records revealed no features suggestive of Cowden syndrome (MIM 158350). Thus, these variants remain of unknown clinical significance.

**Table 1:** Clinical characteristics of the 96 cases with familial colorectal cancer.

Characteristics		
	**Mean (SD)**	**Range**
Age, mean (SD)	70 (11)	32–90
	**Count**	**(%)**
Gender		
Female	45	47%
Male	51	53%
First degree relatives with CRC		
1	86	90%
2	8	8%
3–4	2	2%
MSI status		
MSS	88	92%
MSI	8	8%
Dukes stage		
A–B	61	64%
C–D	34	35%
Histologic grade		
1–2	81	84%
3–4	11	11%
Location*		
Distal	59	61%
Proximal	36	38%

NOTE: some of the numbers do not match due to missing data.

Abbreviations: MSI, microsatellite instability; MSS, microsatellite stable.

* Distal, from splenic flexure to rectum; proximal, from cecum to transverse colon.

### Identification of colorectal cancer predisposing variants

We hypothesized that predisposing germline variants would likely be rare in the general population, and predicted to truncate the protein product. We therefore filtered the data to prioritize such variants (Figure 1). First, variants had to be protein truncating with putative loss-of-function alteration; including nonsense, frameshift (insertion and deletion) or splice-site variants (IVS +1, +2, −1, and −2). A total number of 3,654 truncating variants were found in the exome data. Second, variants were excluded if present in the 1000 Genomes Project [16] or population matched exome control data (n = 212) at minor allele frequency (MAF)>0.001. After control filtering, 2,090 truncating variants remained. Third, genes with truncating variants in more than one familial CRC case were selected for further analysis. There were a total of 588 such variants of which 422 were frameshift, 115 nonsense, and 51 splice site variants (Figure 1). Frameshift variants were grossly overrepresented in the list of truncating variants due to sequencing artifacts. Finally, manual filtering was performed on all variants to further remove artifacts due to duplicated regions, mapping errors, and systematic sequence specific errors. The filtering procedure resulted in a shortlist of 29 genes with 46 truncating variants. These were subsequently validated by Sanger sequencing (Figure 1).

Sanger sequencing was successful for all amplicons, and 23 truncating variants in 18 genes were confirmed. Of these seven were frameshift, 12 nonsense, and four splice-site variants. To further exclude neutral polymorphisms, the confirmed variants were screened in 310 Finnish population matched controls, of which approximately two-thirds were also regionally matched. Variants with MAF>0.001 in the overall discovery phase control set (including Finnish control exome data and Sanger sequenced controls) were excluded (Figure 1).

**Figure 2:**
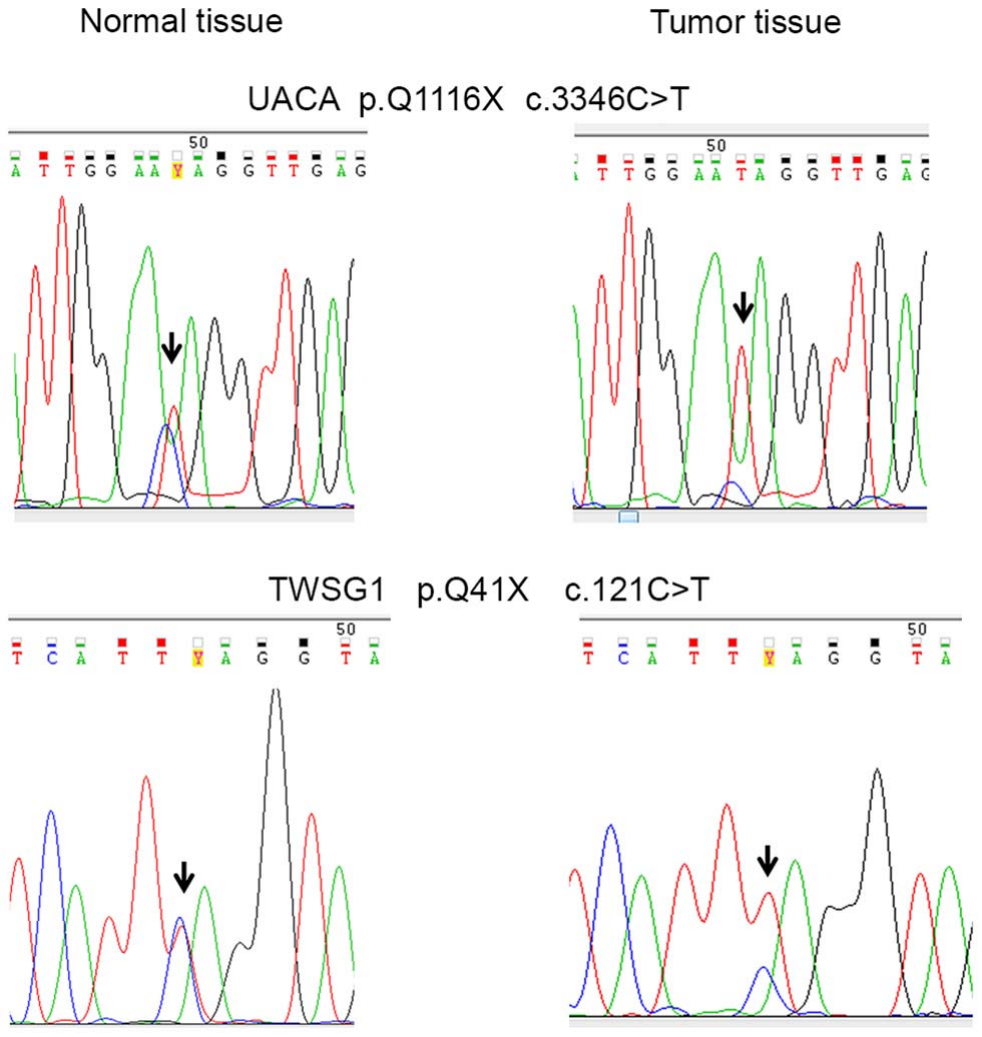
Examples of DNA sequence chromatograms. Chromatograms on the top demonstrate c.3346C>T, p.Q1116X in *UACA*. Chromatograms on the bottom demonstrate c.121C>T, p.Q41X in *TWSG1*. DNA extracted from tumor tissue shows LOH with retention of the mutated alleles (right). The wild-type alleles can still be seen in the tumor chromatograms, due to normal tissue contamination in the tumor samples.

In total, we identified 11 candidate predisposing genes with 14 truncating germline variants in at least two familial CRC cases (Table 2); *UACA*, *SFXN4*, *TWSG1*, *PSPH*, *NUDT7*, *ZNF490*, *PRSS37*, *CCDC18*, *PRADC1*, *MRPL3*, and *AKR1C4*. A summary of all these variants and respective frequencies are presented in Table 2. Gene descriptions and proposed functions of the identified genes are listed in Table S2. Typically, the same truncating variant was detected in several patients. However, three genes harbored two different types of truncating germline variants (Table 2). Nine genes showed truncating variants in 2/96 familial cases. Two genes had truncating variants in 3/96 cases; *UACA (uveal autoantigen with coiled-coil domains and ankyrin repeats)* (3/96, 3.1%) and *SFXN4 (sideroflexin 4)* (3/96, 3.1%). In *UACA*, p.Q1116X was identified in two out of 96 familial cases and present in 522 Finnish population matched controls with a MAF of 0.001. *UACA* p.R1292X was found in one out of 96 cases and the variant was not found in controls (Figure 2). In *SFXN4*, three out of 96 cases had c.32delC. This variant had a MAF of 0.001 in population matched controls. None of the other identified truncating variants were identified in population matched controls, except for c.389_390insA in *PSPH* which was found in 1/502 controls (MAF 0.001). To further explore the frequency of these variants in controls, we referred to the Exome Variant Server (NHLBI GO Exome Sequencing Project (ESP), Seattle, WA, http://evs.gs.washington.edu/EVS/ [July 2013]). Three of the identified germline variants, *SFXN4* c.32delC, *NUDT7* c.111T>A, and *PRSS37* c.176+1G>A, were reported, however, at a MAF of less than 0.0003.

**Table 2:** Candidate colorectal cancer predisposing genes with Sanger validated truncating variants in familial CRC cases.

								VALIDATION PHASE SAMPLES		
Gene	Ensembl Gene	Ensembl Transcript	Chomosomal position	Nuclotide (cDNA)	Amino acid (protein)^a^	Familial cases	Finnish^b^ population matched controls	Finnish population matched cases	Finnish population matched controls	Loss wt allele
*UACA*	ENSG00000137831	ENST00000322954	15:70959677C>T	c.3346C>T	p.Q1116X	2/96	1/522	2/862	1/550	3/4
*UACA*	ENSG00000137831	ENST00000322954	15:70959149C>T	c.3873C>T	p.R1292X	1/96	0/494	1/823	0/550	0/2
*SFXN4*	ENSG00000183605	ENST00000355697	10:120925120delC	c.32delC	fs	3/96	1/502	–	–	0/3
*TWSG1*	ENSG00000128791	ENST00000262120	18:9337345C>T	c.121C>T	p.Q41X	2/96	0/494	0/886	0/545	2/2
*PSPH*	ENSG00000146733	ENST00000275605	7:56084959-56084958insA	c.389_390insA	fs	2/96	1/502	–	–	1/2
*NUDT7*	ENSG00000140876	ENST00000268533	16:77759403T>A	c.111T>A	p.Y37X	2/96	0/494	–	–	0/2
*ZNF490*	ENSG00000188033	ENST00000311437	19:12691841C>T	c.1048C>T	p.R350X	2/96	0/491	1/877	0/551	1/3
*PRSS37*	ENSG00000165076	ENST00000350549	7:141539137G>A	c.176+1G>A	sp	1/96	0/491	–	–	0/1
*PRSS37*	ENSG00000165076	ENST00000350549	7:141537678G>A	c.413G>A	p.W138X	1/96	0/489	–	–	0/1
*CCDC18*	ENSG00000122483	ENST00000370276	1:93682195A>C	c.1878-2A>C	sp	1/96	0/492	–	–	0/1
*CCDC18*	ENSG00000122483	ENST00000370276	1:93712480C>G	c.3325C>G	p.S1109X	1/96	0/475	–	–	0/1
*PRADC1*	ENSG00000135617	ENST00000258083	2:73457240G>A	c.168+1G>A	sp	2/96	0/482	–	–	0/2
*MRPL3*	ENSG00000114686	ENST00000264995	3:131181721A>G	c.895-2A>G	sp	2/96	0/487	–	–	0/2
*AKR1C4*	ENSG00000198610	ENST00000380448	10:5254628delA	c.620delA	fs	2/96	0/491	–	–	0/2

Gene, transcript and chromosomal positions taken from Ensembl build 37 (http://www.ensembl.org).

a fs = frameshift insertion and deletion variant, sp = splice site variant.

b Counts include both exome data controls and Sanger sequenced controls.

The exome data was also searched for missense variants in the 11 candidate predisposition genes; five missense variants were observed in five genes (Table S3). All of the missense variants were present in one case only, except for p.Q83H in *PSPH* which was identified in two out of the 96 familial cases. None of the missense variants were predicted to have a damaging effect on the protein by either of the prediction programs used (Table S3). The identified missense variants were very rare in population matched controls (MAF<0.001).

### Loss of heterozygosity analysis

Loss of heterozygosity (LOH) was examined in cancers of CRC cases with candidate predisposing germline variants (Figure 1). The following genes displayed LOH in at least one cancer: *UACA*, *TWSG1*, *PSPH*, and *ZNF490* (Table 2). Seven LOH events were observed and all targeted the wild-type allele (*P* = 0.0078). In *UACA* three out of six examined tumors showed loss of the wild-type allele and in *TWSG1 (twisted gastrulation protein homolog 1)* both of the tumors showed loss of the wild-type allele (Figure 2).

### Genotyping population matched cases and controls

Variants in genes showing loss of the wild-type allele in tumor tissue were genotyped in an independent set of validation phase samples (Figure 1). This set included 954 Finnish population matched CRC cases and 586 Finnish population matched controls. *UACA* p.Q1116X was identified in two additional unrelated CRC cases and one control (Table 2). The ages at diagnosis were 67 and 58 years for the two cases. In the overall set of Finnish population matched controls used in this study, two out of 1,108 controls had *UACA* p.Q1116X (MAF = 0.0009). *UACA* p.R1292X was found in one additional case (diagnosis at the age of 61) and no controls were heterozygous for this variant. The variant p.R350X in *ZNF490* was found in one additional case (diagnosed at the age of 58) and remained absent in controls (Table 2). *TWSG1* p.Q41X was not present in any additional cases or controls. Genotyping was not successful for *PSPH* c.389_390insA. Next, LOH was analyzed in the tumors of the four additional cases with truncating variants (Table 2). One of the additional cases with *UACA* p.Q1116X showed LOH involving the wild-type allele (Figure 2).

### Segregation

**Figure 3:**
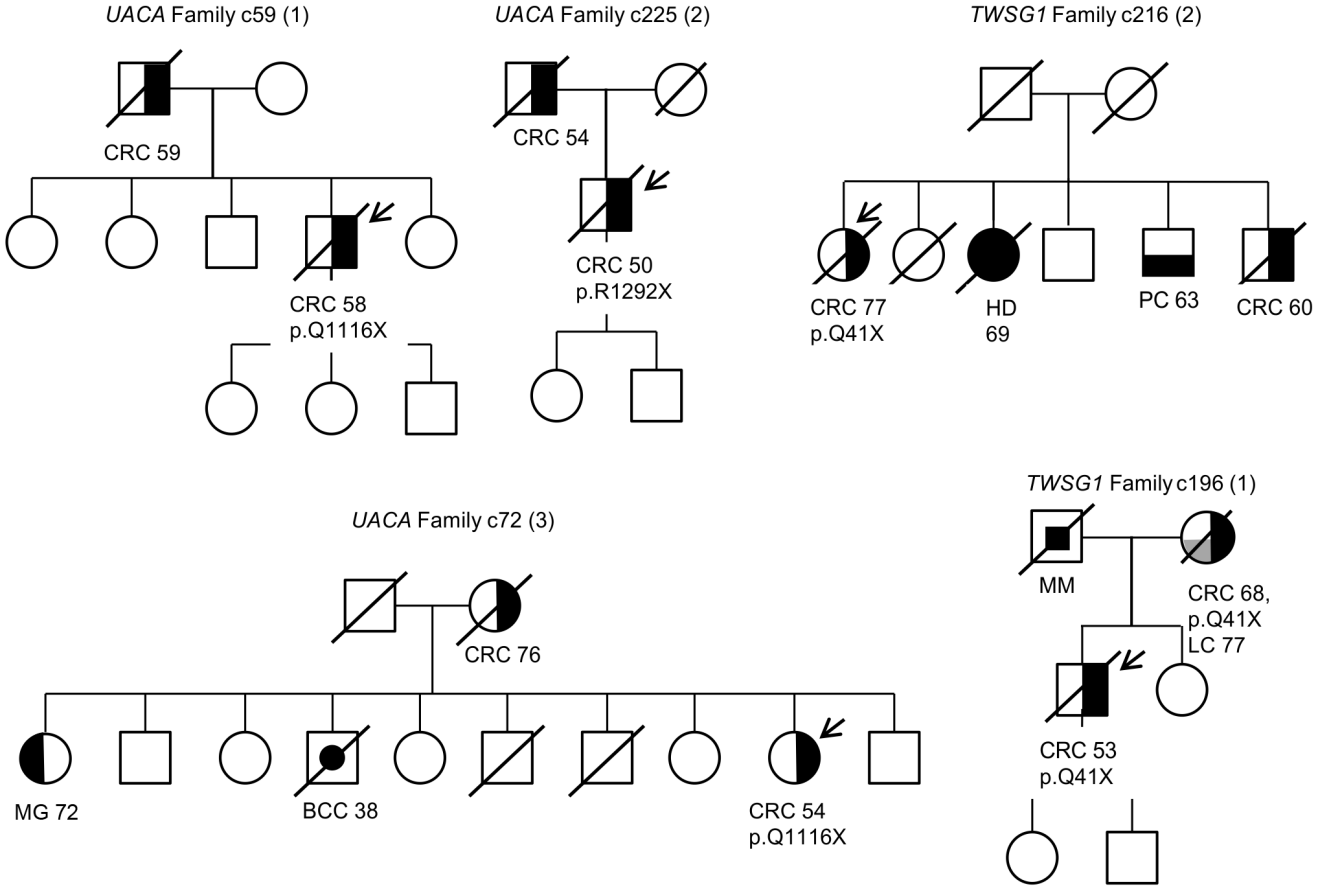
Pedigrees of families found to carry *UACA* and *TWSG1* truncating variants. Carrier status is depicted for all the cases for whom readily extracted DNA was available. The individuals that underwent exome sequencing are marked with an arrow. Numbers represent the age at diagnosis of the affected individuals. The following abbreviations are used: CRC, colorectal cancer; BCC, basal cell carcinoma, MG, meningioma; HD, Hodgkin lymphoma; PC, prostate cancer; MM, melanoma and LC, lung cancer.

Segregation analysis of the identified truncating variants was performed for all the affected first degree relatives for whom samples were available. In total, segregation was analyzed in seven families for five of the identified truncating variants; c.32delC in *SFXN4*, p.Q41X in *TWSG1*, p.R350X in *ZNF490*, c.168+1G>A in *PRADC1*, and c.620delA in *AKR1C4* (Figure 3 and Figure S1). The following variants showed segregation; c.32delC in *SFXN4*, c.168+1G>A in *PRADC1*, and c.620delA in *AKR1C4*. The variant p.Q41X in *TWSG1* segregated in one family but not the other (Figure 3) and p.R350X in *ZNF490* did not segregate (Figure S1).

## Discussion

Exome sequencing is a powerful tool for discovering novel genetic variants that predispose to disease [17]. To examine the genetic basis of common familial CRC we exome sequenced 96 independent cases (Table 1) derived from a previously described population-based collection of patients [4], [18] and from an additional unselected collection (unpublished). To our knowledge, this is the largest effort to date where familial CRC has been studied by exome sequencing to identify novel CRC predisposing genes. Several strategies were applied to improve the power of gene discovery. First, a large set of familial CRC cases (at least one first-degree relative diagnosed with CRC) was utilized, negative for any known high penetrance CRC mutation. Second, the cases were from Finland, known for its isolated population with reduced genetic heterogeneity. Such isolated populations are enriched for rare founder variants, facilitating identification of disease genes [15]. Third, tumor tissue availability for all the CRC cases allowed for the assessment of somatic allelic imbalance, which gave important additional information related to pathogenicity of the variants. Fourth, genotyping of selected variants was performed in a set of validation phase population matched samples, consisting of 954 cases and 586 controls.

In total, we identified 11 novel candidate CRC susceptibility genes with rare truncating variants in two or three familial CRC cases; *UACA*, *SFXN4*, *TWSG1*, *PSPH*, *NUDT7*, *ZNF490*, *PRSS37*, *CCDC18*, *PRADC1*, *MRPL3*, and *AKR1C4* (Table 2 and Table S2). They were absent or rare (MAF≤0.001) in the general population. The results fit with the “rare variant hypothesis” that proposes that a significant proportion of the missing heritability of complex diseases is due to a series of rare variants, each conferring a moderate increase in risk. Typically, such risk alleles function dominantly and independently [19], [20]. The “rare variant hypothesis” is strongly supported by evolutionary theory, which argues that variants that promote disease are selected against and are therefore rare. Another argument for the hypothesis comes from recent empirical population genetic data which shows that rare variants are enriched for deleterious mutations [21]. The question remains whether the identified candidate genes act as classical tumor suppressors with second hits or show alternative characteristics, such as haploinsufficiency or dominant-negative effects. Of the genes identified, four out of 11 showed loss of the wild-type allele in at least one tumor. In total, seven LOH events were observed and none showed loss of the mutant allele (*P* = 0.0078). This suggests that complete inactivation of these genes seems to be preferentially selected for in tumor evolution and that these germline variants are prime candidates for CRC susceptibility.

Perhaps the strongest candidate predisposition gene, in view of the LOH data and case frequency, was the apoptosis-associated gene *UACA*. Three of the 96 familial CRC cases were found to carry heterozygous truncating variants (p.Q1116X and p.R1292X) in *UACA* (Table 2). We performed genotyping to screen the variants in a set of validation phase samples. We identified three additional unrelated cases who were heterozygous for the variants encoding either p.Q1116X or p.R1292X. Second hits by LOH involving the germline wild-type allele were found in three of the six tumors (Figure 2). The average age of onset of CRC in the familial cases was 54 years (58, 54 and 50) (Figure 3), younger than the mean age of onset of 71 in familial cases without the *UACA* truncating variants (Table S1). UACA has recently been identified as a novel regulator of apoptosis. It is known to reside within the Apaf-1/procaspase-9 complex and regulate apoptosis activating factor (APAF-1). It also regulates the apoptotic pathway by controlling the activation of nuclear factor (NF)-κB [22]. In addition, *UACA* gene expression has been shown to be down-regulated in non-small cell lung carcinoma (MIM 211980) [23]. Taken together, the loss of UACA in cancer cells might result in altered activation of apoptotic pathways, ultimately promoting genesis of CRC.

Another gene of particular interest was *TWSG1*. The detected truncating germline variant (p.Q41X) was present in two familial CRC cases (2/96 cases) and completely absent in 1,039 Finnish population matched controls (Table 2). Loss of the remaining normal *TWSG1* allele was observed in both tumors indicating that the gene might act as a classical tumor suppressor gene (Figure 2). The index case with Q41X in family 1 developed CRC at the age of 53 and segregation analysis showed that the variant was inherited from the affected mother (Figure 3). The mother had developed CRC at the age of 68 and lung cancer at the age of 77. The variant did not segregate with CRC in family 2. Rare risk alleles of moderate penetrance are usually over-represented in familial cases; however co-segregation of disease is not always observed [19]. Previous studies have shown TWSG1 to be a regulator of BMP-signaling [24]. It is known to act downstream of TGF-β, inducing SMAD2 phosphorylation and mediating DNA binding on *Smad3/4* consensus sites [25]. TWSG1 functions in cellular pathways that are essential in genesis of CRC, however, its exact role in these pathways remains to be clarified.

In summary, exome sequencing is a well-justified strategy for discovering cancer predisposing variants. The identification of predisposing variants has substantial implications for disease risk assessment and surveillance in family members. Here, we identified eleven candidate predisposing genes with truncating variations in familial CRC. A key challenge is how to identify predisposing variants in the background of non-pathogenic polymorphisms. Screening the eleven genes in familial CRC cases representing different populations will be important to gain robust evidence for pathogenicity, as well as to characterize the natural history of the respective phenotypes. This information, then, can be translated into tools for cancer prevention and early diagnosis in individuals carrying true predisposition alleles.

## Materials and Methods

### Samples

This study was reviewed and approved by the Ethics Committee of the Hospital district of Helsinki and Uusimaa (HUS). Signed informed consent or authorization from the National Supervisory Authority for Welfare and Health was obtained for all the study participants.

#### Discovery phase samples

A population-based material of normal and tumor tissue from 1,042 CRC patients was collected between 1994 and 1998 from nine Finnish central hospitals [4], [18]. After 1998 sample collection continued from two of the nine hospitals (unpublished collection). From the additional series 472 CRCs and respective normal tissue samples were available. From these materials, familial CRC cases were selected according to the following criteria: (i) at least one CRC case in a first degree relative, (ii) negative for any known high penetrance CRC mutation, and (iii) availability of sufficient amount of DNA extracted from normal tissue (Figure 1). As part of previous efforts [4], [18], all cases had been tested for MSI, and in positive cases Sanger sequencing of *MLH1* and *MSH2* had been performed. Known polyposis syndromes could be excluded for all the familial cases based on medical and pathological reports. In total, 96 familial cases fulfilled the above mentioned criteria. Clinical characteristics are presented in Table 1 and in more detail in Table S1. Both germline DNA extracted from blood or normal colonic tissue and corresponding fresh-frozen tumour DNA were available. Information on histological tumor grade and Dukes stage was obtained from pathology reports. The discovery phase control set compromised the following non-overlapping collections; in-house Finnish population matched control exome data (n = 212) and DNA samples (n = 310) from population matched healthy individuals obtained from the Finnish Red Cross Blood Transfusion Service. Around two-thirds of the control DNA samples were regionally matched controls.

#### Validation phase samples

Finnish population matched cases (n = 954) were selected from a population-based material of 1,042 CRC patients [4], [18] and from an additional series of 472 Finnish CRC cases, based on DNA availability. The control DNA samples (n = 586) were from population matched healthy individuals and obtained from the Finnish Red Cross Blood Transfusion Service.

### Exome sequencing

The Agilent SureSelect Human All Exon Kit v1 (Agilent, Santa Clara, CA, USA) was used to capture exomic regions. Paired end short reads were sequenced on either Illumina GAII or HiSeq platform (Illumina Inc., San Diego, CA, USA). Raw sequence data was received in FASTQ format and quality checked with FASTQC (http://www.bioinformatics.bbsrc.ac.uk/projects/fastqc). All exomes passed the quality control. 3′ ends with high adapter similarity were removed by an in-house script whereafter reads were mapped to the human reference genome GRCh37 by BWA (Burrow-Wheelers Aligner). Duplicates were removed with Picard Tools (http://picard.sourceforge.net) MarkDuplicates. Local realignment was done by Genome Analysis Toolkit (GATK) IndelRealigner to improve the detection of small insertions and deletions. The initial single nucleotide variant (SNV) and indel calls needed for creating the GATK realignment intervals were made using samtools mpileup and downloaded from the 1000 genomes project Phase I indel calls ([16]the August 2010 release). Final SNV and indel calls were made using the GATK UnifiedGenotyper with a low variant quality score threshold (1.0).

### Exome data analysis

Exome data analysis was performed in “Rikurator”, an in-house visualization and comparative analysis tool (unpublished). The tool allowed for simultaneous analysis of all the 96 exomes and interactive quality/control filtering. The following quality filters were used: (i) variants had to have a quality score ≥50, (ii) coverage had to be ≥6, and (iii) the percentage of mutated reads had to be ≥30. Truncating variants, including nonsense, frameshifting insertion and deletion, or splice-site alteration IVS +1, +2, −1, and −2, were extracted. Data was control filtered against population matched exome control data (n = 212) and data from the 1000 Genomes Project (Phase 1 release) [16]. Variants were excluded if present in the 1000 Genomes Project or exome control data at MAF>0.001 (Figure 1). Genes with truncating variants present in at least 2/96 cases were studied further. Manual filtering was performed on all variants to further remove artifacts due to duplicated regions, mapping errors, and systematic errors. Systematic errors, both position specific and sequence specific, in high-throughput sequence data have been described previously by Meacham et al [26]. Finally, outputs were generated for Ensembl canonical transcripts (Ensembl build 37).

### Sanger validation of the exome findings

Potential loss-of-function variants were verified by Sanger sequencing from DNA extracted from normal tissue samples. Sequencing primers were designed with the Primer3-program (http://frodo.wi.mit.edu/primer3/) using NCBI37/Hg19 as the reference sequence. The primer sequences can be found in Text S1. The fragments were amplified with the AmpliTaqGold enzyme (Applied Biosystems, Foster City, CA). The PCR products were purified using the ExoSAP-IT PCR purification kit (USB Corporation, Cleveland, OH, USA). Electrophoresis was run on a 3730xl DNA Analyzer (Applied Biosystems at Institute for Molecular Medicine Finland, FIMM). The sequencing reactions were performed utilizing the Big Dye Terminator v.3.1 kit (Applied Biosystems, Foster City, USA), Sanger sequencing was performed implementing the ABI3100×l technology (Applied Biosystems), and the sequence graphs were visualized with the Chromas – software (version 2.33, Technelysium Pty Ltd, Helensvale, Australia). The results were analyzed both manually and with the Mutation Surveyor –software (version v3,30, Softgenetics, State College, PA, USA). Confirmed truncating variants were Sanger sequenced in 310 Finnish population matched healthy controls, of whom about two-thirds were regionally matched. Sanger sequencing was performed as described above. All variants that had a MAF>0.001 in the discovery phase control set were excluded.

### Loss of heterozygosity analysis

Sanger sequencing was also performed on DNA extracted from tumor tissue in cases carrying validated truncating variants. All tumors had been microscopically evaluated by a pathologist and all except one contained ≥50% of carcinoma tissue. Loss of heterozygosity was analyzed by comparing allelic ratios of tumor and respective normal tissue DNA, as previously described [27]. Peak heights were manually measured from sequence graphs based on which allelic ratios were calculated.

### Genotyping of population matched cases and controls

Variants in genes showing loss of the wild-type allele in tumor tissue were genotyped in a set of validation phase samples, comprising 954 population matched CRC cases and 586 population matched controls. Genotyping was carried out by using the 7900HT Fast Real-Time PCR System (Applied Biosystems) and was performed at the Estonian Genome Center, University of Tartu. The variant p.Q41X in *TWSG1* was genotyped using massARRAY iPLEX Gold (Sequenom, San Diego, CA) and performed at the Institute for Molecular Medicine Finland (FIMM), University of Helsinki. The genotyping conditions and primers utilized can be found in Text S1. Genotyping success rates were over 90% for all the variants, except for *PSPH* where the genotyping assay failed. All the variants identified by genotyping were further confirmed by Sanger sequencing.

### Missense variants at candidate CRC loci

The exome data was searched for missense variants at the 11 candidate predisposition loci. The same filtering criteria were utilized as for truncating variants. The variants were excluded if present in Exome Variant Server (NHLBI GO Exome Sequencing Project (ESP), Seattle, WA, http://evs.gs.washington.edu/EVS/ [July 2013]) with MAF>0.001. The functional effects of the identified missense variants were predicted by SIFT (http://sift.jcvi.org/) and PolyPhen 2 (http://genetics.bwh.harvard.edu/pph2/).

### Segregation of truncating variants

Archived Formalin-fixed, Paraffin-embedded (FFPE) tissue samples were ordered for first degree relatives with CRC whenever possible. Genomic DNA was extracted from all available FFPE samples. Sanger sequencing was performed on identified truncating variants to test for segregation. In total, segregation was analyzed in seven families for five of the identified truncating variants.

### Statistics

One-tailed exact binominal test was used for *P*-value calculations.

## Supporting Information

Figure S1 Pedigrees of families with truncating variants in *ZNF490*, *SFXN4*, *PRADC1*, and *AKRIC4*, in which segregation analysis was carried out. The individuals that underwent exome sequencing are marked with an arrow. Carrier status is depicted for all the CRC cases. Numbers represent the age at diagnosis of the affected individuals. The following abbreviations are used: CRC, colorectal cancer; BCC, basal cell carcinoma; GC, gastric cancer; G, glioma; HD, hodgkin lymphoma; PC, prostate cancer; MM, melanoma and LC, lung cancer.
(TIF)

Table S1 Clinical information on the 96 familial colorectal cancer cases exome sequenced.
(XLS)

Table S2 Gene description and proposed gene function of the identified candidate CRC predisposing genes.
(XLS)

Table S3 Missense variants at candidate CRC predisposition loci.
(DOC)

Text S1 PCR primers utilized in the Sanger validation of the variants, genotyping conditions and primers utilized in genotyping.
(DOCX)
