# Supplementary figures and images for: Eleven Candidate Susceptibility Genes for Common Familial Colorectal Cancer
Source: PLoS Genet. 2013 Oct 17;9(10):e1003876. doi: 10.1371/journal.pgen.1003876 (PMC3798264; doi:10.1371/journal.pgen.1003876)

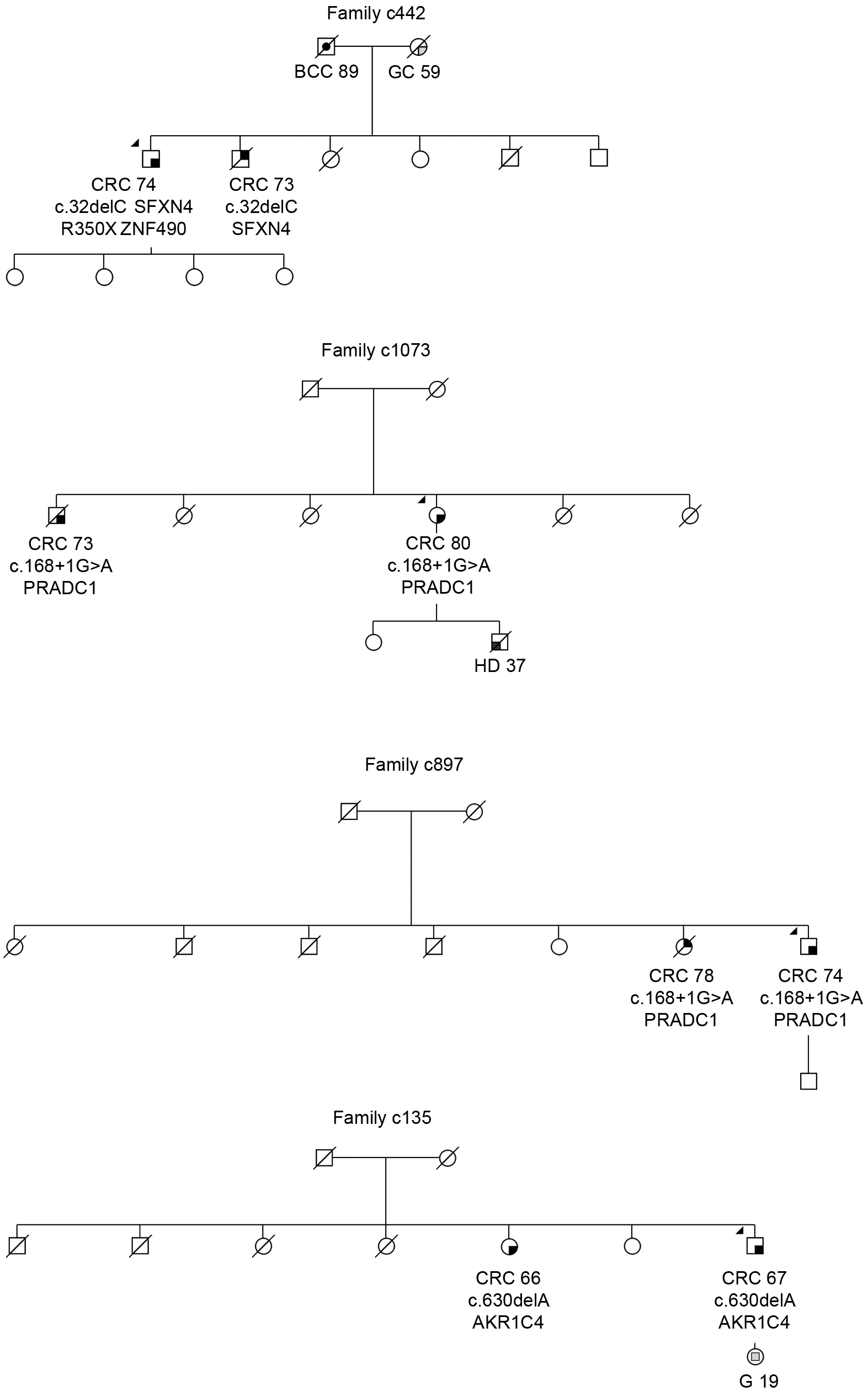

Supplement: Figure S1 — Pedigrees of families with truncating variants in ZNF490, SFXN4, PRADC1, and AKRIC4, in which segregation analysis was carried out. The individuals that underwent exome sequencing are marked with an arrow. Carrier status is depicted for all the CRC cases. Numbers represent the age at diagnosis of the affected individuals. The following abbreviations are used: CRC, colorectal cancer; BCC, basal cell carcinoma; GC, gastric cancer; G, glioma; HD, hodgkin lymphoma; PC, prostate cancer; MM, melanoma and LC, lung cancer. (TIF) [file pgen.1003876.s001.tif]
