# Supplementary material for: Eleven Candidate Susceptibility Genes for Common Familial Colorectal Cancer
Source: PLoS Genet. 2013 Oct 17;9(10):e1003876. doi: 10.1371/journal.pgen.1003876 (PMC3798264; doi:10.1371/journal.pgen.1003876)
Supplement: Table S3 — Missense variants at candidate CRC predisposition loci. (DOC) [file pgen.1003876.s004.doc]

|  | | | | | | | |
| --- | --- | --- | --- | --- | --- | --- | --- |
| **Gene** | **Ensembl Gene** | **Ensembl Transcript** | **Chomosomal position** | **Amino acid (protein)** | **Polyphen Prediction** | **Sift Prediction** | **Cases** |
| *UACA* | ENSG00000137831 | ENST00000322954 | 15:70961161G>A | p.A621V | possibly damaging | tolerated | c897 |
| *CCDC18* | ENSG00000122483 | ENST00000370276 | 1:93672855G>C | p.R424P | probably damaging | tolerated | c665 |
| *MRPL3* | ENSG00000114686 | ENST00000264995 | 3:131206533G>A | p.T207I | benign | tolerated | c581 |
| *PSPH* | ENSG00000146733 | ENST00000395471 | 7:56087319T>G | p.Q83H | benign | tolerated | c456, c265 |
| *AKR1C4* | ENSG00000198610 | ENST00000380448 | 10:1685615401G>A | p.R66Q | possibly damaging | tolerated | c352 |
| Gene, transcript and chromosomal positions taken from Ensembl build 37 (http://www.ensembl.org) | | | | | | | |
