# Supplementary material for: Eleven Candidate Susceptibility Genes for Common Familial Colorectal Cancer
Source: PLoS Genet. 2013 Oct 17;9(10):e1003876. doi: 10.1371/journal.pgen.1003876 (PMC3798264; doi:10.1371/journal.pgen.1003876)
Supplement: Text S1 — PCR primers utilized in the Sanger validation of the variants, genotyping conditions and primers utilized in genotyping. (DOCX) [file pgen.1003876.s005.docx]

**SUPPLEMENTARY INFORMATION**

**PCR primer sequences utilized**

| AKR1C4_6-1_F | TGTTTTGAATTATCTGATGCTTTTC |
| --- | --- |
| AKR1C4_6-1_R | TGCTCTTATTACCATAGTTTATGTCG |
| AKR1C4_6-1_F_1paraffin | CAAATTTAATGTTATACTTTATTCAGC |
| AKR1C4_6-1_R_1paraffin | GTTGGGTTCCCAGAGCAC |
| AKR1C4_6-1_F_2paraffin | TCTGATGCTTTTCTCTCTTGA |
| AKR1C4_6-1_R_2paraffin | GGCATTTTAGGTAAACTTCCTG |
| AKR1C4_6-2_F | AATTGGCCATGTGCACTTCT |
| AKR1C4_6-2_R | CATAGTTTATGTCGTTGGGTTCC |
| CCDC18_13-1_F | ACTTTGAAAGAGGAGCAGGTGT |
| CCDC18_13-1_R | GCTCCTGTTCTAGTTCTGCATTT |
| CCDC18_23-1_F | TTTTAGGTTACACATTTGGATATGACT |
| CCDC18_23-1_R | AGGGGTGTTTTTAGTTACAGTTTGA |
| MRPL3_10-1_F | TTCACATTAGAAAATGTAGTAGCGAGT |
| MRPL3_10-1_R | TCCTCTTCATCTCCATCAGGA |
| NUDT7_2-1_F | GCTTGCTAAAATGTCTGTCTGG |
| NUDT7_2-1_R | TGAGGTTTAAGGGCAGGAAA |
| PRADC1_2-1_F | CTTCAGGCTTCCGTATCCAT |
| PRADC1_2-1_R | GTGGGATGAGAGGGTCACTG |
| PRADC1_2-1_F_paraffin | GCTGAGTCCTGGGGACATT |
| PRADC1_2-1_R_paraffin | GTGGGATGAGAGGGTCACTG |
| PRSS37_2-1_F | ACCTCAAGTCTCACTTCAACCC |
| PRSS37_2-1_R | ACATTGCCAAGTGTACCCTGT |
| PRSS37_3-1_F | CATGCTCAATCCCAAAGTCC |
| PRSS37_3-1_R | TCCTCCTCTCTCCTCACAGC |
| PSPH_3-1_F | TTTGCAGGGAGCTGGTAAGT |
| PSPH_3-1_R | CCCAGAATACTGAACAATGGAA |
| SFXN4_1-1_F | GTTCCGATAGTGACCCCTTG |
| SFXN4_1-1_R | GTACTTGGCGCTCGGTGAT |
| SFXN4_1-1_F_1paraffin | CCGGCTCCTCCTCCAC |
| SFXN4_1-1_R_1paraffin | CCCGGGCCGTACTTGG |
| SFXN4_1-1_R_2paraffin | CCCTCCCTGCCCCTAGT |
| SFXN4_1-2_F | GTTCCGATAGTGACCCTTG |
| SFXN4_1-2_R | GTACTTGGCGCTCGGTGAT |
| TWSG1_2_F | ACCCTAGCACTTGCCTTTGA |
| TWSG1_2_R | TCCAGACCTGTGTGTCATGATT |
| TWSG1_2-1_F | TCCTGATGTTCCTGACATGG |
| TWSG1_2-1_R | TCCAGACCTGTGTGTCATGATT |
| TWSG1_3_F | TGCAAATATGGCAGGGTTTT |
| TWSG1_3_R | CTTCCCAAGGATAAGCACTTT |
| TWSG1_4_F | GGTAGGAAAATATCCATGTGTAATCC |
| TWSG1_4_R | CAAAAGGCTGCTCCAAAAGA |
| TWSG1_5_F | GCCCTTTGCCTTCTTCAGAT |
| TWSG1_5_R | GGGATTCTGATACAAGATACAACCA |
| UACA_16-1_F | GAAGCTAGTAGAAGAAAATGCCAAA |
| UACA_16-1_R | CTGTCTGCTGCTCTTTCTCG |
| UACA_16-2_F | GCATGCCAAAAAGAAGGAAA |
| UACA_16-2_R | CCGTGCAGACTACTTTGTACCT |
| UACA_16-3_F | TTTGCATGCCAAAAGAGG |
| UACA_16-3_R | TTTGTTTAGCAGATTCTTGTATTCTTC |
| UACA_16-4_F | GTAGAAGAAAATGCCAAACAGAC |
| UACA_16-4_R | CTCTGGGGTTTCCAACACAT |
| ZNF490_5-1_F | GGGAAAGCCTTCAGTTGTCC |
| ZNF490_5-1_R | TCTTTCGTGCACTTCACAGG |
| ZNF490_5-1_F_paraffin | CTTACGGAGTCATGAGAAAACTC |
| ZNF490_5-1_R_paraffin | AAGGCTTCCCCACATTTCTT |

**Genotyping conditions for UACA, PSPH and ZNF490**

The DNA sample concentrations were measured by a Nanodrop ND-8000 spectrophotometer. Dilutions and sample aliquoting as well as assay distribution was carried out on a Hamilton MicroLab StarPlus liquid handling robot. The DNA aliquoting volume was 3,5 ul and the sample concentration ranged from 2 to 5 ng/ul. The aliquoted DNA was dried overnight at room temperature and 4 ul of 1X assay solution was added to each sample and negative control. The assay solutions were prepared using 40X Applied Biosystems TaqMan® SNP Genotyping Assays and Termo Scientific Maxima Probe/ROX qPCR Master Mix (2X) (product no #K0233).

PCR thermal cycling was performed on an MJ Research PTC-225 Tetrad PCR System according to the assay manufacturers recommended protocol - initial hold of 10 min at 95°C, 40 cycles of 15 sec denaturing at 92°C and 1 min extending at 60°C, and a final stage of 2 min at 50°C.

The post-PCR plate reads were done on an Applied Biosystems 7900HT Fast Real-Time PCR system, and the data analysis was performed on the systems SDS 2.4 software. The alleles were called by hand.

| **Primer sequences used in genotyping** | |  |
| --- | --- | --- |
| **Gene and variant** | **Forward primer sequence** | **Reverse primer sequence** |
| UACA_3346C_T | CTGAGATACTTGCAGTGCAAAATCTTT | GTTCTTCCTTTAGATTTTCAATTGTGCCATT |
| UACA_3873C_T | CTGCATTTCAGCATTGAGCAAGAA | TGTATTCTTCTTTGTAACTCTGTGATTGTTGT |
| PSPH_389_390insA | GAGCATGTTGCTTCAAAGCTCAATA | CATCTTACCGTTAAAGTAGAATTTCAGCCT |
| ZNF490_1048C_T | GAATGTGGGAGAGCCTTCTTTTCT | AACTCCGGTGTGGGTTTTCA |

**Genotyping conditions for TWSG1**

Genotyping was performed using the Sequenom MassARRAY system and the iPLEX Gold assays (Sequenom Inc., San Diego, USA). In this method allele discrimination is based on primer extension with single mass-modified nucleotides followed by MALDI-TOF mass spectrometry. All reactions are performed in multiplexes of up to 35 SNPs.

Genotyping reactions were performed on 20 ng of dried genomic DNA in 384-well plates according to manufacturer’s recommendations and with their reagents. Both PCR and extension primers were designed using MassARRAY Assay Design software (Sequenom). Concentrations of the extension primers were adjusted according to their mass and varied between 7-24.6 μM. The data was collected using the MassARRAY Compact System (Sequenom) and the genotypes were called using TyperAnalyzer software (Sequenom). For quality control reasons, the genotype calls were also checked manually. Genotyping quality was examined by a detailed QC procedure consisting of success rate checks, duplicated samples, water controls and Hardy-Weinberg Equilibrium (HWE) testing.

| **Primer sequences used in genotyping** | |  |
| --- | --- | --- |
| **Gene and variant** | **Forward primer sequence** | **Reverse primer sequence** |
| TWSG1_121C_T | ACGTTGGATGGCTAGTGATGTGAGCAAATG | ACGTTGGATGCTGCTCTACAATATATTTTG |
